# High resolution microfluidic assay and probabilistic modeling reveal cooperation between T cells in tumor killing

**DOI:** 10.1038/s41467-022-30575-2

**Published:** 2022-06-03

**Authors:** Gustave Ronteix, Shreyansh Jain, Christelle Angely, Marine Cazaux, Roxana Khazen, Philippe Bousso, Charles N. Baroud

**Affiliations:** 1 Physical microfluidics and Bioengineering, Institut Pasteur, Univerité Paris Cité, 25–28 Rue du Dr. Roux, Paris, France; 2 LadHyX, CNRS, École Polytechnique, Institut Polytechnique de Paris, 91120 Palaiseau, France; 3 Dynamics of Immune Responses Unit, Equipe Labellisée Ligue Contre le Cancer, Institut Pasteur, Université Paris Cité, INSERM U1223, 75015 Paris, France

**Keywords:** Lab-on-a-chip, Multicellular systems, Biological physics, Immunological surveillance

## Abstract

Cytotoxic T cells are important components of natural anti-tumor immunity and are harnessed in tumor immunotherapies. Immune responses to tumors and immune therapy outcomes largely vary among individuals, but very few studies examine the contribution of intrinsic behavior of the T cells to this heterogeneity. Here we show the development of a microfluidic-based in vitro method to track the outcome of antigen-specific T cell activity on many individual cancer spheroids simultaneously at high spatiotemporal resolution, which we call Multiscale Immuno-Oncology on-Chip System (MIOCS). By combining parallel measurements of T cell behaviors and tumor fates with probabilistic modeling, we establish that the first recruited T cells initiate a positive feedback loop to accelerate further recruitment to the spheroid. We also provide evidence that cooperation between T cells on the spheroid during the killing phase facilitates tumor destruction. Thus, we propose that both T cell accumulation and killing function rely on collective behaviors rather than simply reflecting the sum of individual T cell activities, and the possibility to track many replicates of immune cell-tumor interactions with the level of detail our system provides may contribute to our understanding of immune response heterogeneity.

## Abstract (web summary)

Anti-cancer cytotoxic T cell responses largely vary among individuals. Here authors show, by stochastic modeling on high throughput T cell behavior and matched tumor spheroid fate data generated by a microfluidics system, that tumor killing is dependent on T cell cooperativity, which might contribute to the heterogeneity of T cell responses.

## Introduction

The capacity of cytotoxic T lymphocytes (CTL) to eliminate tumor cells is the basis for the development of important tumor immunotherapies such as immune checkpoint inhibitors (e.g., anti-CTLA-4, anti-PD1, or anti-PD-L1 mAbs) and the development of cellular therapies such as CAR T cells^1,2^. However, patient response to these therapies can be highly variable. While many parameters are known to influence patient response to immunotherapies, the number, phenotype, and distribution of CTLs can have a strong predictive value in several types of cancer^3^.

These observations underscore the need to better understand how a successful T cell attack proceeds and what are the critical parameters associated with CTL behavior and function that favor tumor regression. In this respect, several key questions remain unanswered. For example, how do CTLs encounter tumor cells and accumulate within the tumor microenvironment (TME)? What are the dynamics of CTL killing and what CTL density is needed for tumor eradication? Are individual CTLs acting autonomously in the TME or do they interact together? Understanding the basic principles that dictate whether a tumor mass is regressing or not is in fact essential to design, optimize, and evaluate tumor immunotherapeutic strategies.

Multiple approaches are available to evaluate T cell cytotoxicity against tumors. In vitro assays in cell suspension have been used to measure CTL killing capacity and, when performed at the single-cell level, provide information on the extent of functional heterogeneity within a T cell population^4^. These in vitro assays, however, lack the complexity of the 3D tumor microenvironment, which strongly impacts T cell behavior and function. At the other end of the spectrum, intravital imaging offers direct insights into the dynamics, signaling, and killing behavior of single T cells within a developing tumor^5–8^. Limitations of these approaches, however, include the fact that they provide a view of the interactions in a limited spatial and temporal window. Indeed continuous observation periods are generally limited to a few hours, precluding a full understanding of T cell histories in the TME.

An interesting emerging platform comes from advanced in vitro models that recapitulate some aspects of the TME while providing access to the system dynamics^9,10^. These include organoids^11,12^, where cells are allowed to organize in three dimensions (3D), or organ-on-a-chip devices^13^, where the microfluidic device represents the organ geometry and the microfluidics enable temporal control of the flows and physical conditions. Recent work has also dealt with combining the advantages of both approaches to produce organoids-on-a-chip^14^. Gathering general rules from these systems that would explain the outcome of a CTL attack remains challenging as it requires to link quantitative measurements of T cell behavior, which is inherently stochastic, with tumor cell fate^9,14^.

Here, we introduce a microfluidic-based approach for the multiplexed analysis of tumor spheroid fate in the presence of defined immune cell populations. This methodology is based on the parallel formation, manipulation, and observation of hundreds of tumor spheroids within stationary microfluidic droplets. When associated with mathematical models, the quantity and quality of spatiotemporally resolved data allow us to pinpoint key behaviors leading to spheroid destruction and to detect and understand heterogeneity of tumor outcomes.

## Results

### Parallelized immune challenge on an integrated microfluidic chip

**Fig. 1:**
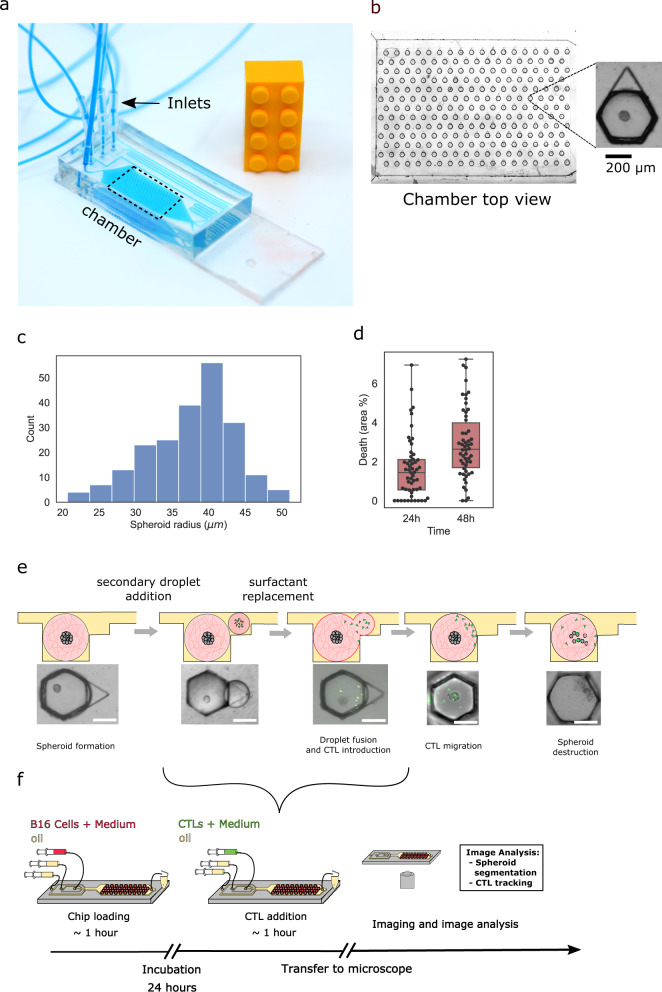
Microfluidic immuno-oncology chip and protocol. **a** Microfluidic chip on a standard glass slide. **b** Expanded view of the trapping region of the chip (dashed box) showing an array of 234 trapped droplets. Each droplet contains a single B16 spheroid in Matrigel, as shown in the inset. **c** Distribution of spheroid radii within a single chip (*N* = 215). **d** Viability measurements using live-dead staining after 24 and 48 h (*N* = 54). **e** Schematic showing a primary droplet with a tumor spheroid, followed by the addition and fusion of a secondary droplet containing GFP-labeled CTLs, eventually leading to tumor cell killing and spheroid fragmentation. Scale bar is 200 μm. **f** Schematic representation of the complete experimental protocol.

The immunogenic rejection of 3D cancer models is studied using the classic model of Ovalbumin-expressing mouse B16 melanoma (B16-OVA), which are challenged by OVA-specific CD8+ cytotoxic T lymphocytes (CTLs) bearing the OT-1 transgenic TCR (see “Methods”)^15–19^. Further, the results obtained with B16-OVA are compared with non-OVA expressing B16 Wild-Type cells (B16-WT) as a control. These two cell types were previously reported to exhibit similar proliferation dynamics and do not differ in their in vivo immunogenicity^18^. The experiments rely on a microfluidic device that consists of a droplet generating region followed by a droplet trapping region that serves to culture the cells and observe them (Fig. 1a, b)^20,21^. This trapping region is patterned with 234 microfluidic anchors^22^, that allow the droplets to be held in place even in the presence of an external flow (see “Methods”). The anchors are diamond-shaped (see Fig. 1b) in order to allow for multiple droplet pairings^23^.

The experiment begins by producing aqueous droplets (volume = 50 nl) containing Matrigel and a suspension of B16 cells at a concentration of 1.5 × 10^6^ cell/ml. To obtain single spheroids in each droplet, a concentration of 2.0 mg/ml of Matrigel was used, with higher Matrigel concentrations leading to the formation of multiple spheroids per droplet (Supplementary Fig. 1a) (see “Methods”). Once the droplets are anchored, the device is placed in an incubator at 37^ ∘^C overnight, which allows a single B16 spheroid to form in each droplet (Fig. 1b). At these cell and Matrigel concentrations we obtain spheroid radii in most cases ranging between 35 to 45 μm (Fig. 1c). A live-dead staining shows that less than 3% of the cells were dead after 48 h in the chip (Fig. 1d).

After overnight incubation, the CTLs are brought to the Matrigel droplets by generating a group of secondary smaller droplets (volume = 10 nL) that contain a broad distribution of CTLs (Supplementary Fig. 1b). These secondary droplets are trapped in the triangular sections of the anchors and then merged with the spheroid-containing Matrigel, thus bringing the two cell populations into the same Matrigel droplet (Fig. 1e)^23^. The interactions between the CTLs and spheroids are observed by time-lapse microscopy, typically over 24 h. The whole process from spheroid preparation to CTL addition and imaging is done on a single chip (Fig. 1f) and each experiment yields up to 234 individual replicates, of which we typically obtain 50 time-lapse movies, due to the small image acquisition time-intervals (2min/frame). For higher time-intervals, more data points can be collected from the same chip. The movies are then analyzed using our custom-made scripts (see Supplementary Software 1).

Three stages in the cell–cell interactions can be identified: the CTL exploration of the gel, their accumulation on the spheroid, and the killing of B16 Ova-expressing cells (see Supplementary Movie 1 for representative cases). These stages are studied in detail below.

### CTL migration in micro-device reproduces in vivo behavior

**Fig. 2:**
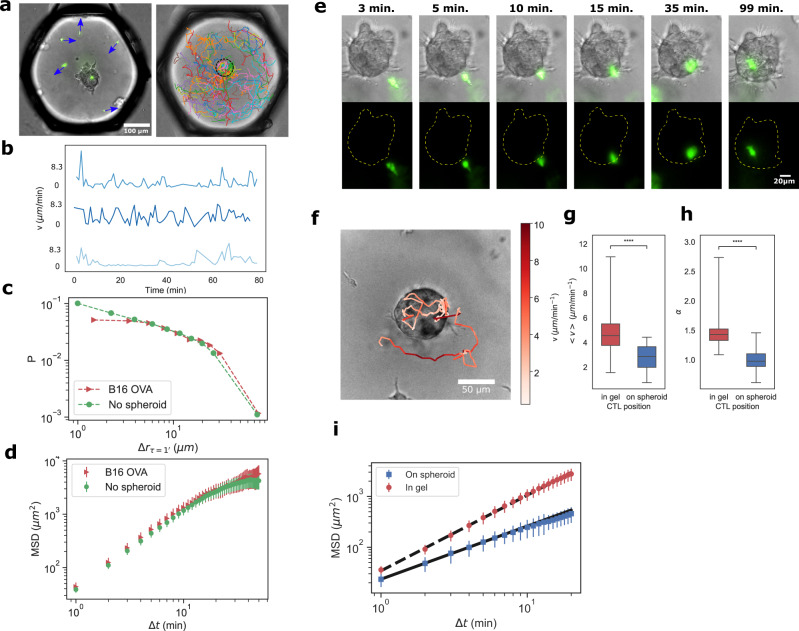
CTL migration in droplets recapitulates in vivo behavior. **a** (left) Representative image of CTLs with instantaneous velocity vectors inside Matrigel droplet. (right) CTL tracks in one droplet over 24 h, each color represents an individual cell track. The dashed black circle outlines the spheroid boundary. **b** Representative velocities as a function of time for three different T cells. **c** Probability distribution of a cell to migrate by a given distance (Δ*r*) during a fixed time step Δ*t* = 1 min (*n* = 67965 points without spheroid and *n* = 34072 individual points for CTLs in presence of the B16 spheroids). **d** Mean-square displacement (MSD) of CTL migration with (*N* = 20 droplets) and without (*N* = 26 droplets) spheroids. Error bars represent the SEM. **e** Time sequence showing the initial CTL approach and contact with a spheroid. **f** Track of a single CTL as it migrates in the matrigel and on the spheroid surface. Colormap represents the instantaneous velocity of the cell. **g**, **h** Average velocity and mean square displacement exponent (*α*) of cells migrating in the gel and on the spheroid. Each data point is the average velocity in a given droplet (*N*_gel_ = 55, *N*_spheroid_ = 54, respective *p*-values of 1.3 × 10^−10^ and 1.2 × 10^−15^). **i** Mean-square displacement of cells migrating in the matrigel and on the spheroid. Bold and dashed lines represent the best fits for the MSD of CTLs on the spheroid and in the matrigel, with respective exponents of 1.1 and 1.4 (measurement conducted over *N* = 54 droplets). Error bars represent the SEM. Source data are provided as a Source data file.

The CTL migration is tracked in the time-lapse movies as the cells perform 3D migration within the gel or on the surface of the spheroid (see Fig. 2a). The recorded velocities display alternating periods of motility and arrest phases, as seen by the high and low velocities in Fig. 2b. This behavior, as well as the value of the velocities, correspond well to previously reported CTL velocities in collagen gels in vitro^24^ or within tissues in vivo^25,26^. We can infer the 3D motility properties from the acquired microscopy data (see “Methods”). Note that the value of the measured velocities depends on the image acquisition frequency (see Supplementary Fig. 2), so we maintain the sampling at 2min/frame for all the experiments (see “Methods”).

In order to evaluate the influence of the spheroid presence in the droplet on the motility of the CTLs, the migration statistics without a spheroid present are compared with the statistics in the presence of a spheroid during the first 500 min of an experiment. We restrict the analysis to cells that are not in contact with the spheroid. The displacement distributions (Fig. 2c) and the mean-square displacements (MSD) (Fig. 2d) do not show any significant difference between the two conditions. In both cases, the CTLs undergo super-diffusive random walks (Fig. 2d) with MSD ~ *τ*^*α*^, where *τ* is the time between two observations and *α* = 1.6, in agreement with what was reported in vitro and in vivo^24,26^.

After some time, one CTL comes in contact with the spheroid. This contact generally leads the T cell to adhere to the spheroid and explore its surface over the course of a few hours (Fig. 2e, f and Supplementary Movie 2). We select individual tracks with segments both on and off the spheroid to investigate more precisely the CTL motility change upon reaching the spheroid. We observe that the CTL behavior is strongly modified: They display lower mean velocity (Fig. 2g and Supplementary Movie 2) with a lower MSD exponent (Fig. 2h, i). The average MSD exponent goes from 1.4 when the cells move in the gel to 1.1 after the same cells have reached the spheroid.

The CTL migration in the gel therefore recapitulates behaviors that have been reported in vivo^24–27^, with the current data highlighting the switch in motility before and after the CTL contacts the spheroid surface.

### A positive feedback loop drives CTL accumulation on the spheroid

**Fig. 3:**
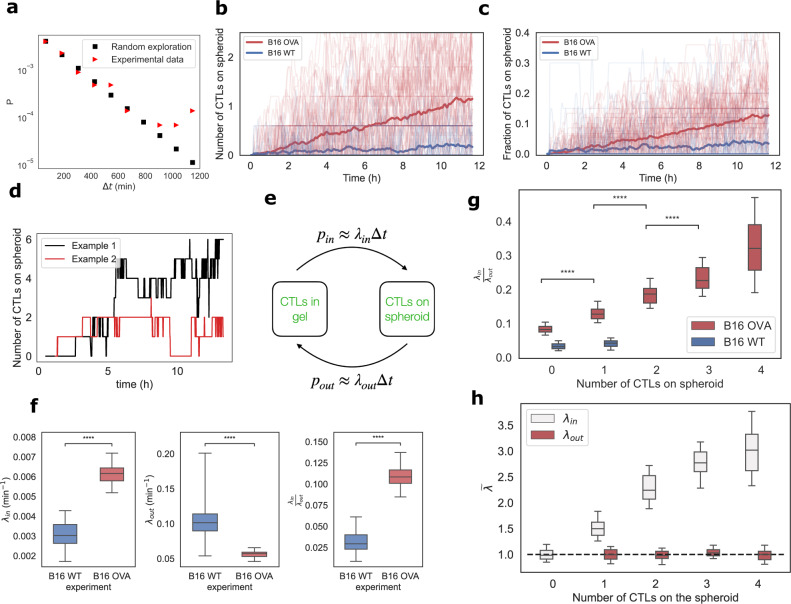
CTL accumulation is  enhanced by a positive feedback-loop after first contact. **a** Experimental distribution of first CTL-spheroid contact times and theoretical distribution for randomly migrating CTLs. **b** Number of CTLs detected on each spheroid and **c** the fraction per droplet as a function of time. Each thin line represents a single tracked spheroid, in bold is the averaged value. In red is the accumulation for B16-OVA spheroids and in blue for B16 WT spheroids [84 individual B16-OVA spheroids and 81 B16 WT spheroids tracked]. **d** Number of CTLs as a function of time on two representative spheroids showing the detection of attachment/detachment events. **e** Schematic of the stochastic accumulation model: CTLs can switch from the gel to the spheroid with different probabilities. *p*_in_(Δ*t*) (conv. *p*_out_(Δ*t*)) is the probability for a cell to attach to (conv. detach from) the spheroid during a time interval Δ*t*. Counting attachment and detachment events in the experiments allow us to infer the rates *λ*_in_ and *λ*_out_. **f** Estimates for the attachment rates (*λ*_in_), detachment rates (*λ*_out_), and affinity ratio (*λ*_in_/*λ*_out_) for B16-WT (blue) and B16-OVA (red) cells. The box plots are obtained by using a bootstrapping method with 50 repetitions as described in the methods (respective *p*-values of 7 × 10^−18^, 1.2 × 10^−16^, and 7 × 10^−18^). **g** The affinity ratio as a function of the number of CTLs detected on the spheroid for B16 WT and B16 Ova spheroids (respective p-values of 6 × 10^−9^, 3 × 10^−15^, and 1.3 × 10^−13^). **h** Normalized attachment rate *λ*_in_ (white) and detachment rate *λ*_out_ (red) as a function of the number of CTLs attached to the spheroid. *λ*_in_ and *λ*_out_ are normalized by their mean values for 0 and 1 CTL on the spheroid, respectively. Source data are provided as a Source data file.

We now investigate the contact time statistics of the CTLs on the spheroids. The distribution of first-passage times is consistent with the distribution of randomly migrating particles in an enclosed environment, indicating that the initial contact is random and that there is no attraction from the spheroid on the CTLs (Fig. 3a). After the initial CTL contact with the B16 cells, the arrival of successive CTLs leads to an accumulation of T cells on the spheroid in the case of the B16-OVA spheroids. This accumulation is shown in absolute numbers (Fig. 3b) and also by computing the ratio of CTLs in each droplet that are present on the spheroid as a function of time (Fig. 3c). However, the accumulation is not observed in the case of wild-type B16 cells, which do not express Ova (Supplementary Movie 3).

At this stage, an important question is whether this accumulation results from cells reaching the spheroid randomly, as they explore the droplet volume, or if the accumulation rate is enhanced due to cell–cell signaling. We address this question by analyzing the accumulation of the CTLs at the spheroid level. At this scale, the CTL accumulation is not homogeneous but stochastic, with CTLs both attaching and detaching over time (Fig. 3d, Supplementary Movie 2, Supplementary Movie 4). A time series of the number of CTLs present on each spheroid is obtained by counting the CTLs that are detected within the spheroid region on the images, with a 2 min time resolution. The statistics of these time series on all of the parallel realizations are then analyzed using a Markov chain method (see “Methods”). This in turn yields the attachment rate *λ*_*in*_ and detachment rate *λ*_out_ for each individual CTL (Fig. 3e).

The value of *λ*_in_ is found to be significantly higher in the B16-OVA spheroids compared the B16-WT spheroids, while the opposite is true for *λ*_out_ (Fig. 3f). These measurements indicate that the arrival rate of CTLs increases when the cells composing the spheroid express the cognate antigen recognized by the CTLs and that they stay attached for longer periods of time. Therefore the accumulation of CTLs on the spheroids is mediated by two independent phenomena: first the increase in arrival frequency and second by the decreased leaving frequency. The net effect of the attachment/detachment dynamics can then be summarized by the affinity ratio, *λ*_in_/*λ*_out_, which accounts for the net effective accumulation of CTLs on target. This ratio is found to be significantly higher with B16-OVA spheroids when compared to the B16-WT spheroids (Fig. 3f).

Evidence of a positive feedback loop for the attraction among the CTLs can be obtained by calculating the change of the affinity ratio, defined as λ_in_/λ_out_, as a function of the number of T cells present on the spheroid. Indeed, the depth of the experimental data allows us to obtain a value of *λ*_in_/*λ*_out_ before the first contact and then successively track the change of this ratio after every contact, in each droplet (Fig. 3g). Again, the data for B16-OVA show a significant difference with the WT case. More interestingly, the higher the number of CTLs present on the B16-OVA spheroid, the higher the ratio, and thereby, the faster the accumulation rate: this is a hallmark of a positive feedback. The increase in the accumulation rate is driven by an increase in *λ*_in_ for a constant value of *λ*_out_ (Fig. 3h). It demonstrates that the accelerated accumulation is mediated by the increasing attraction of the CTLs to the spheroid, which starts from the very first CTL attached to the spheroid (Fig. 3h).

The current results confirm recently published observations^15^ that show a CCR5-mediated swarming of T cells in vitro, as well as in vivo studies that report the accumulation of T cells on targets^28,29^. Figure 3 shows that these effects occur even for cell populations consisting of a few individuals and that a single contact can trigger the beginning of the positive feedback. Below we go beyond the CTL accumulation to address the relationship between the accumulation of CTLs and their capacity to kill the B16 spheroids.

### Killing of B16 cells by CTLs is heterogeneous

**Fig. 4:**
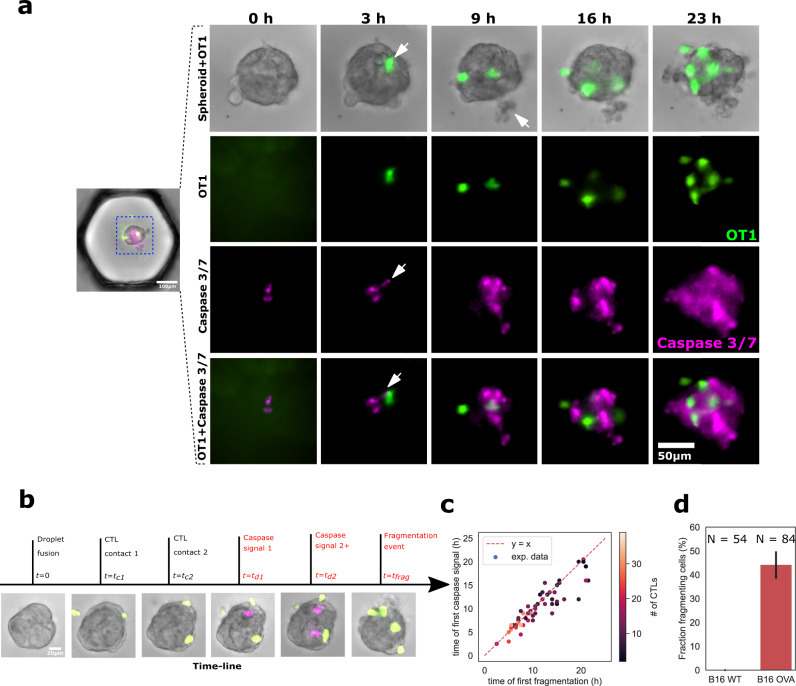
CTLs kill tumor cells within spheroids. **a** Representative sequence showing CTL (OT1) positions on the spheroid, caspase 3/7 fluorescent death marker, and B16 cell fragmentation. The white arrow at 3h indicates the appearance of capsase signal next to a CTL. At 9 h it represents a fragmented dead cell. **b** Representative chronology showing the key events for a given spheroid interaction with CTLs: Contact times of CTLs on spheroids, detection of caspase 3/7 signal, detection of fragmentation events. **c** Time of first caspase signal vs. first observation of cell fragmentation. **d** Percentage of WT (black) and OVA (red) spheroids that show at least one fragmentation event in under 14 h. *N* equals 54 and 84 spheroids, respectively. The error bar is the SEM. Source data are provided as a Source data file.

After focusing on the behavior of the T cells we now turn to the response of the cancer spheroids upon CTL accumulation. The time-lapse movies allow us to identify individual cell death events in the spheroids at the molecular level by detecting the activation of Caspase 3/7, which provides an early marker of apoptosis^30^ (Fig. 4a and Supplementary Movie 5). In the brightfield image, we observe instances of rapid shedding of cellular material and debris from the spheroids (Fig. 4a, Supplementary Movies 1, 3, 5), which we refer to as “spheroid fragmentation”. Combining this information with the position of CTLs relative to the spheroid, it is thus possible to record a detailed chronology of the key events taking place in each droplet by timing successive CTL contacts with the spheroid and the apparition of fragmentation events and Caspase 3/7 signals, as shown in Fig. 4b.

We observe that the timing of the first Caspase event post-CTL contact is well correlated with the first fragmentation event (Fig. 4c), indicating that the two observations are closely related. For this reason, we will hereafter use brightfield images to quantify spheroid killing, which simplifies the analysis pipeline. Furthermore, the timing of these killing events is highly variable, ranging from a few hours to beyond 24 h. For some spheroids, no fragmentation or Caspase events are observed over the course of an experiment. In the following analysis, we will label “successful killing” the cases when the first fragmentation event is observed before *t* = 14 h. The statistics of such events are summarized in Fig. 4d, which shows that 44% (*N*_OVA_ = 84 spheroids) of the OVA expressing spheroids are successfully killed by the CTLs. This contrasts with the B16-WT spheroids, where we do not observe any fragmentation events (Fig. 4d, Supplementary Movie 3). An analysis of the statistical impact of each of the problem parameters on a successful killing shows that the dominant parameters are the number of CTLs in the droplet and the number of CTLs that reach the spheroid.

### Tumor spheroid killing involves collective effects

We now consider the relationship between the spatiotemporal dynamics of the CTLs and the tumor spheroid outcomes (successful or unsuccessful killing). An indication of the relevance of this link is obtained first by observing that the CTL accumulation rates are faster on the spheroids that display fragmentation than in the opposite case, both in absolute numbers (Supplementary Fig. 3a) and as a fraction of the total number of cells per droplet (Supplementary Fig. 3b). This indicates that a faster accumulation is correlated with efficient killing.

**Fig. 5:**
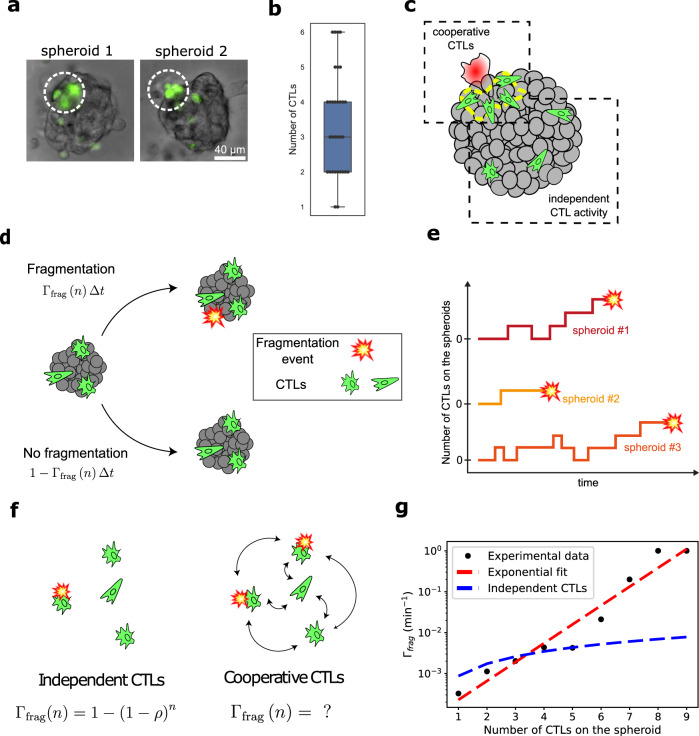
CTL number and collective behavior determine probability of killing. **a** Two representative images showing CTL clustering on the spheroid during first fragmentation event. The white circles have a radius of 30 μm around fragmenting cell. **b** Distribution of the CTL numbers within a radius ≤30 μm around the fragmentation areas (*N* = 31). **c** Sketch summarizing the observed trends: CTLs (green) migrate on the surface of the spheroid and cluster together in particular regions, where fragmentation of B16 cells (red) is observed. **d** During a time-interval Δ*t*, a spheroid with *n* CTLs attached to it has a probability Γ_frag_Δ*t* of fragmenting. **e** Illustration of possible scenarios of the number of CTLs on the spheroids and the apparition of fragmentation. **f** The fragmentation rate Γ_frag_ can be modeled as the result of independent CTLs interacting with the spheroid, with an individual fragmentation rate per CTL worth *ρ*. Conversely, the fragmentation rate can be viewed as the result of a collaborative process. **g** Estimates of Γ_frag_ as a function of the number of CTLs on the spheroid *n*. Experimental measurements (*N* = 84, black dots) are fitted with an exponential (dashed red line) compared with the results of the independent CTL model (dashed blue line). Source data are provided as a Source data file.

Moreover observing the CTLs on the spheroid reveals that fragmentation events are associated with the presence of several CTLs in the vicinity (Fig. 5a, b). This local effect is quantified by counting the number of T cells present within a 30 μm radius of the first cell fragmentation event, giving a mean number of 3.4 cells (median at 3 cells), with fragmentation very rarely occurring with only one cell present at the fragmentation site (Fig. 5b). Indeed, CTLs sometimes appear to besiege a salient B16 cell, causing it to burst after a few minutes of attack. This suggests that the CTLs tend to cluster at particular sites on the spheroid and that their clustering enhances their ability to induce spheroid fragmentation (Fig. 5c).

The above observations can be analyzed in greater depth to demonstrate that the cooperative effect of the CTLs enables the killing of tumor cells. Here, we first infer the fragmentation probability of cancer cells as a function of the number of CTLs present on the spheroid (*n*). If we consider a time-interval of length Δ*t*, the probability of a cancer cell fragmentation event happening is Γ_frag_(*n*)Δ*t* and conversely, the probability of the cell not fragmenting is 1 − Γ_frag_(*n*)Δ*t* (Fig. 5d) (see “Methods”). Comparing the different droplets we see that the fragmentation events can happen at different time-points as is schematically represented in Fig. 5e. From the experimentally observed fragmentation times Γ_frag_ can be inferred: ${{{\Gamma }}}_{{{{frag}}}}(n)=\frac{{K}_{{{{Death}}},n}}{{K}_{{{{Death}}},n}+{K}_{n}}$. Here *K*_Death,*n*_ is the number of fragmentation occurrences with exactly *n* CTLs on the spheroid and *K*_*n*_ the number of instances with *n* CTLs on the spheroid before the first fragmentation event.

The experimental measurements are compared with a probabilistic model where the CTLs behave independently from each other as is schematically represented in Fig. 5f (see “Methods”). This independent model leads to the parabolic probability of killing (blue line in Fig. 5g) which poorly matches with the experimental data. On the other hand, the experimental evolution of Γ_frag_ is well-fitted by an exponential increase (red dashed line), demonstrating that a model of independent interactions between the CTLs and the target cells fails to capture the underlying mechanism of spheroid fragmentation.

### Long and short-range interactions combine to determine probability of CTLs to kill a tumor spheroid

**Fig. 6:**
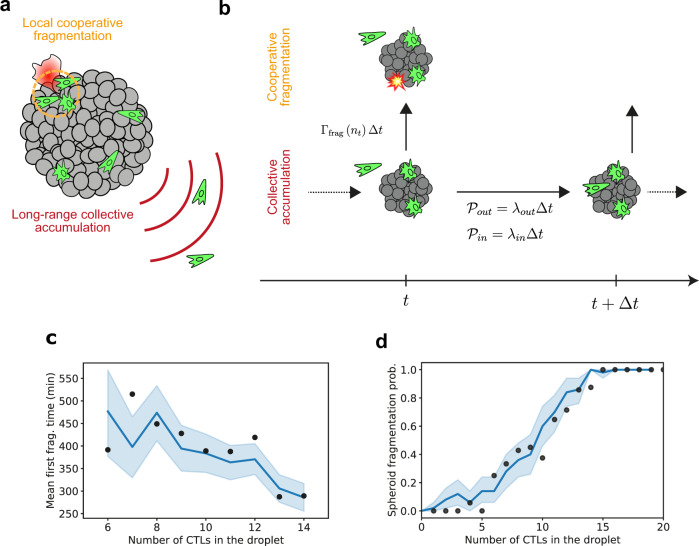
Combining short and long-range interactions to simulate spheroid fate. **a** The concentration-dependent killing is modeled as the result of two complementary mechanisms: long-distance cooperative attraction of CTLs to the target site and local killing cooperation on the spheroid. **b** The evolution of the spheroid fate in the droplets can be modeled as a branching process: at each time step the spheroid can fragment with a probability Γ_frag_Δ*t* or not, and CTLs can either attach to or detach from the spheroid. **c** Simulations of the spheroid fate using the model in (**b**) and parameters obtained above recover the experimentally derived first-fragmentation times. Shaded area represents the 95% confidence interval of simulated data. The bold line represents the mean. **d** Experimental (black dots) and simulated (blue line) spheroid fragmentation probability as a function of the number of CTLs in the droplet. Shaded area represents the 95% confidence interval of simulated data. The bold line represents the mean. Source data are provided as a Source data file.

The probability for successful killing to occur for any particular spheroid can now be explained as a combination of the effect of CTL accumulation on the spheroid and their cooperative killing behavior (Fig 6a). This probability is a function of the total number of CTLs in the droplet, which also correlates with the maximum number observed on the spheroid (see Supplementary Fig. 4 and Table 1). The spheroid fate can be simulated as a two-step branching process. First, we simulate the evolution of the number of CTLs on the spheroid at each time step based on the experimentally derived parameters *λ*_in_ and *λ*_out_, shown in Fig. 3. Second, we account for the possible spheroid fragmentation depending on the number of CTLs on the spheroid and Γ_frag_, as described in Fig. 5. We then repeat the process for each time-interval, updating the number of T cells on the spheroid and the spheroid state (fragmented or intact) iteratively until the time of the simulated experiment has lapsed. The model process is illustrated in Fig. 6b (see “Methods”).

**Table 1:** Generalized linear model (GLM)^42^ results (number of samples is 96) from the statsmodel API^45^.

Variable	Coeff. *β*	Std err	*z*	*p*	[0.025	0.975]
*n*	1.84	0.786	2.342	0.019	0.300	3.381
*N*	1.5567	0.707	2.203	0.028	0.172	2.942
Area	−0.2175	0.320	−0.681	0.496	−0.844	0.409
*t*_1_	−0.5405	0.999	−0.541	0.588	−2.498	1.417
*t*_2_	1.1746	1.732	0.678	0.498	−2.221	4.570
*t*_3_	−2.3092	2.635	−0.877	0.381	−7.473	2.854
*t*_4_	1.4984	2.191	0.684	0.494	−2.795	5.792

*n* is the maximum recorded number of CTLs on the spheroid and *N* is the number of T cells detected in the droplet, Area is the spheroid surface and *t*_1_ to *t*_4_ are the first times at which 1 to 4 CTLs are detected on the spheroid surface.

This computation is performed for a number of CTLs per droplet (*N*) ranging from 0 to 20 cells and repeated 50 times for each value of *N*. This allows us to first compare the experimental first fragmentation times with the simulated ones (Fig. 6c). In both cases, the first fragmentation time decreases as the number of CTLs increases in the droplet and the simulated values closely fit the experimentally observed fragmentation times. We then compare the simulated fragmentation probability curve as a function of the number of CTLs in the droplet to the experimental data (Fig. 6d). Not only does the simulation confirm the key role of CTL number in causing spheroid fragmentation, but the close match between simulated and experimental measurements indicates that the spheroid fragmentation process is well recapitulated from these two mechanisms: collective accumulation and cooperative killing of CTLs at the spheroid site.

## Discussion

The current study introduces a new paradigm for extracting biological information from in vitro experiments, by treating the parallel realizations as *“Monte-Carlo experiments*” that reach different outcomes in a probabilistic way. This contrasts with existing microfluidic models for cancer-immune interactions, which treat each chip as a single experiment and use traditional biological measurement techniques^10,31^. By comparison the droplet format provides several unique features, including the ability to merge many droplet pairs at a well-defined time, thus providing a common starting time of the parallel experiments^23,32^. Moreover, the encapsulation within droplets allows the conditions in each of the parallel experiments to be well controlled, thus allowing for massively multiplexed experiments on a single device.

Here these technical advantages are associated with probabilistic modeling to infer key biological information about the ability of CTLs to sense and respond to the tumor, by relating the spatiotemporal dynamics of the CTLs with the outcome for the tumor spheroid. Specifically, we find that the first CTL-tumor cell contact, which occurs randomly, triggers a positive feedback loop that leads to an accelerated accumulation of CTLs on the spheroid. Later, CTLs form clusters on the spheroid that enhance their ability to kill the target cells, leading to tumor rejection.

Several mechanisms may account for the collaborative CTL accumulation and killing. Chemokines that are both sensed and produced by T cells have the ability to drive their swarming behavior^15^. Cooperative killing, in which multiple sublethal cytotoxic hits synergize to induce target cell killing, has been previously described in the context of viral infection^33^ and tumor development^17^. Alternatively, initial CTL-tumor cell interactions may facilitate tumor destruction by increasing MHC class I expression through IFN-*γ* production and diffusion in the tumor microenvironment for example^25,34^. As illustrated here, our approach helps support and generate new hypotheses that can be subsequently dissected at the molecular level.

Looking ahead, the current platform can now be generalized to include several immune cell types and more realistic tumor models in each droplet. Here again the spatiotemporal resolution and *Monte-Carlo* approach will be fundamental to understand the causality of the interactions and the effect of 3D geometry. Finally, working with patient-derived organoids^35,36^ will have important implications for personalized medicine.

## Methods

### Experiments and analysis

#### Statistics and reproducibility

The box plots represent the quartiles of the distribution in all figures where this data representation method is used (i.e., Figs. 1d, 2g, h, 3f–h, and 4b) as detailed in the Seaborn library documentation^37^. The whiskers describe the distribution minima and maxima.

The statistical tests used to compare distributions are Mann–Whitney–Wilcoxon tests with two-sided Bonferroni corrections. For all figures concerned (i.e., Figs. 2g, h and 3f, g) we have the following mapping for *p*-value annotations: ns: 5.00 × 10^−2^ < *p* ≤ 1; *: 1.00 × 10^−2^ < *p* ≤ 5.00 × 10^−2^; **: 1.00 × 10^−3^ < *p* ≤ 1.00 × 10^−2^; ***: 1.00 × 10^−4^ < *p* ≤ 1.00 × 10^−3^; ****: *p* ≤ 1.00 × 10^−4^.

One representative image from a data set of total 84 droplets in the case of B16-Ova and 54 in the case of B16-WT has been included in Figs. 1e and 2a, e, f, respectively. A representative image sequence from a total of 61 droplets has been used for Fig. 4a, b, respectively. One representative image from a total of 200 droplets each for aqueous, 2.025 and 4.05 mg/ml condition is being used for Supplementary Fig. 1a.

The data used to generate Figs. 2d, i, 3g, h, and 6c, d are pooled from three different microfluidic chips. Each droplet in these experiments corresponds to a unique experimental condition (cell number, spheroid size, spatial distribution of cells, initial condition of the T cells, etc.). The stochastic models then provide values of the parameters *λ* and Γ_frag_ by tracking the co-evolution of different cellular events within each droplet. As such each droplet in each microfluidic device should be treated as an independent replicate of the stochastic evolution. The bootstrapping analysis of the statistics confirms that the use of different subsets of these data does not modify the results.

#### Tumor cells

B16-WT melanoma and B16-OVAlbumin peptide (residues 257–264) expressing cell-lines (B16-OVA) were maintained in RPMI 1640 (Fischer Scientific - 12027599) media containing 10% FBS and 1% penicillin-streptomycin antibiotics and were maintained at 5% CO_2_ and 37 ^∘^C. B16.F0 (B16) and B16.F0-Ova (B16-OVA) melanoma cells were kindly provided by Claude Leclerc.

#### Generation of OVA-specific cytotoxic T lymphocytes (CTLs)

Ubi-GFP Rag1−/−OT-1 TCR mice were bred in our animal facility under specific pathogen-free conditions. The mice were bred and managed by Institut Pasteur’s animal facility with a central air conditioning equipment which maintains constant temperature of 22 ± 2 ^∘^C. Air is renewed at least 20 times per hour in animal rooms. Fluorescent light is provided with a 14:10-h light:dark cycle. Humidity is monitored but not controlled and in the range of 25–65%. Splenocytes were isolated from Ubi-GFP OT-1 TCR transgenic mice and red blood cells were removed by ammonium-chloride-potassium lysis. One-third of the cells was then pulsed with 50 μM of Ova257-264 peptide (SIINFEKL) for 2 h at 37 ^∘^C in 1 mL total volume of RPMI medium 1640-GlutaMAX. The rest of the cells were incubated at 37 ^∘^C in 15 mL of complete medium (RPMI medium 1640-GlutaMAX supplemented with 10% heat-inactivated fetal bovine serum, 50 μg/mL penicillin, 50 μg/mL streptomycin, 1 mM sodium pyruvate, 10 mM HEPES, and 50 μM *β*-mercaptoethanol). After 2 h, the two populations were mixed and cultured for 3 days. Cells were then subjected to Ficoll gradient centrifugation to remove dead cells and thus select live Ova-specific CTLs, and cultured in complete RPMI medium, supplemented with human IL-2 (10 ng/mL; R&D) for 2 additional days. All animal studies were approved by the Institut Pasteur Safety Committee in accordance with French and European guidelines (CETEA 2017-0038).

#### Microfabrication, microfluidic setup, and droplet formation

The PDMS-based microfluidic device on which the experiments were conducted is precisely described in refs. ^20,23,38^. Preceding droplet production, the chip in filled with fluorinated FC40 (3M) oil mixed with 2%(v/v) FluoroSurfactant (Ran Biotechnologies) and cooled at −20^ ∘^C for 2 h to prevent Matrigel gelification during the loading. The primary droplets are produced as in ref. ^20^. The aquous phase is composed of RPMI media, Corning Matrigel (Dutscher Dominique - 354234) and B16 melanoma cells at a concentration of 1.5 × 10^6^ cells/mL. The primary droplets have a volume of ca 50 nL. The chip is then placed in the incubator at 37 ^∘^C leading to Matrigel gelification.

The secondary droplets (volume ca. 10 nL) were produced with an aqueous phase containing RPMI media and OT-1-CD8+GFP cells at a concentration of 1.5 × 10^6^ cells/mL. The secondary droplets were further trapped in the triangular regions adjacent to the individual hexagonal wells already present with primary droplets encapsulated with B16 spheroids (see Fig. 1). To fuse the primary with the secondary droplets, 20% (v/v) of 1H,1H,2H,2H-perfluoro-1-octanol (PFO) (Sigma-Aldrich) was dissolved in NovecTM-7500 Engineered Fluid (3M) and was perfused in the microfluidic chip. This caused the fusion of adjacent droplets. After the fusion of droplets, a fresh solution of FC40 and Fluorosurfactant was flushed-in to remove the PFO in the microfluidic chamber.

#### Spheroid formation in different matrigel concentrations

We primarily used the Matrigel concentration of 2.0 mg/ml in order to encapsulate the droplets with B16 cells. Twenty-four hours after droplet loading, the B16 cells self-assemble into a single 3D spheroid of B16 tumor cells (Fig. 1b). Using concentrations higher than 2.0 mg/ml (4.05 mg/ml) often leads to multiple spheroids (Supplementary Fig. 1a) located at different droplet heights. Conversely, droplets with pure aqueous media (without Matrigel) resulted in single spheroid formation but lacked the Extra-cellular Matrix (ECM) necessary for the migration of T cells (Supplementary Fig. 1a).

#### Viability assay (Fig. 1)

B16-OVA spheroid-containing droplets were made according to the protocol described above with the addition of Propidium Iodide (PI) (Sigma - P4864) at a concentration of 3 μM. The chip was then imaged at 24 and 48 h after seeding. Only spheroids positioned in the center of the droplets were imaged, in order to avoid artifacts due to the microscopy. Using a custom-made Imagej macro, the area of red fluorescent signal by PI was measured and the percentage fluorescent area was calculated when compared to the complete spheroid area.

#### Apoptosis assay (Fig. 4)

Caspase-3/7 Red Apoptosis Assay Reagent (Essen Bioscience - 4704) was used at 2 μM concentration (added during primary droplet formation) in order to visualize the apoptotic cells in the spheroids.

#### Microscope imaging

Images were captured using a Nikon Ti2 motorized epifluorescence microscope with a ×20 objective lens. The illumination was produced by a Lumencor LED light source and the images were captured by a Hamamatsu C13440-20CU SCMOS camera. Raw data collection was done using imaging software Nikon Elements (version 5.11.01, Build 1367).

#### Image analysis

A specific image analysis pipeline was developed in order to extract physical and biological variables from the time-lapse movies. The routines were written in Python (version 3) programming language and use several open-source libraries^39–41^. The code is available on the GitHub repository associated to this publication. Basic data visualization and quantification of spheroid size distribution is done using custom-made macro in Imagej (version 1.53 f51).

#### Determining the positions of CTLs with regard to the spheroid

Each droplet image is multi-channel consisting in brightfield and FITC (510 nm) channels. The former enables well identification and spheroid segmentation, whereas the latter enables us to record CTL positions.

To segment the spheroid in a given droplet we rely upon a border-detection routine based on Laplace filtering. The spheroid properties such as size and position are recorded and stored. This procedure is repeated at every time step for more robustness. The CTLs are detected using the fluorescence channel, and their positions are stored. This information is then crossed with the spheroid positions extracted above to determine the position of the CTLs relative to the spheroid in the droplet (on/off the spheroid). For each time-step an image with the raw image, the mask covering the detected spheroid and the relative positions of the CTLs is generated for manual verification a-posteriori of the algorithm efficacy. Faulty wells (with for example mis-detected spheroids) are then thrown away before data analysis. The full image-analysis algorithm is available on the GitHub repository associated with this publication.

#### *λ*_in_ and *λ*_out_ estimation and simulation

The attachment and detachment rates are estimated using a bootstrapping procedure. From a subset of the experimental accumulation plots (Fig. 3d) we count the attachment and detachment events and estimate the values of *λ*_in_ and *λ*_out_. We repeat the procedure 50 times selecting for each iteration 70% of the total number of spheroids in each condition (84 B16-OVA and 81 B16-WT spheroids). This gives us a distribution of the estimated parameters *λ*_in_ and *λ*_out_ represented in Fig. 3. The code necessary for this estimation is available on the GitHub repository associated with this publication.

### Mathematical models

#### Interpreting the statistics of cell displacements from 2D images

After the introduction of the T cells into the Matrigel droplets, they explore the space of the droplet in all three dimensions (3D). For practical considerations, however, the imaging was limited to a single plane within the droplet, leading to two-dimensional (2D) slices of the drops. It is therefore important to consider how to interpret the statistics of 2D measurements of cells moving in 3D. Under the hypothesis of a perfectly isotropic medium, we can study the influence of 2D projection of a 3D motion on the mean square displacement (MSD). For a displacement during a time-interval *τ*, we can write the following relationship for the displacement vector **r**:

1 $${ \langle {\parallel} {{{{{{{\bf{r}}}}}}}}{\parallel} \rangle }_{\tau }={\left\langle \sqrt{{r}_{x}^{2}+{r}_{y}^{2}+{r}_{z}^{2}}\right\rangle }_{\tau }=\sqrt{3}{ \langle | {r}_{k}| \rangle }_{\tau },$$

where *r*_*i*_ is the displacement in the direction *i* and $\langle \cdot \rangle$ denotes an ensemble average. Therefore, we should have:

2 $${ \langle {\parallel} {{{{{{{{\bf{r}}}}}}}}}_{p}{\parallel} \rangle }_{\tau }={\left\langle \sqrt{{r}_{x}^{2}+{r}_{y}^{2}}\right\rangle }_{\tau }=\sqrt{\frac{2}{3}}{ \langle | {{{{{{{\bf{r}}}}}}}}{\parallel} \rangle }_{\tau },$$

Correcting the velocity values from experimental 2D measurements yield estimated 3D velocities on the order of 6.3 μm/min, which are in line with observations in vivo^25^.

Also, the scaling of the mean square displacement as a function of *τ* is not impacted by the projection from 2D to 3D as demonstrated by the calculation below:

3 $${ \langle {{\parallel}} {{{{{{{{\bf{r}}}}}}}}}_{p}{{\parallel}}^{2} \rangle }_{\tau }={ \langle {\parallel} {{{{{{{{\bf{r}}}}}}}}}_{x}{{\parallel} }^{2}+{\parallel} {{{{{{{{\bf{r}}}}}}}}}_{y}{{\parallel} }^{2} \rangle }_{\tau }=\frac{2}{3}{ \langle {\parallel} {{{{{{{\bf{r}}}}}}}}{{\parallel} }^{2} \rangle }_{\tau }$$

The calculation also shows that the effective diffusion coefficient *D*_*p*_ of a three-dimensional track estimated from a 2D measurement is worth 2/3 of the “true” diffusion coefficient *D*.

#### CTL velocity measurements depend on the imaging frame rate

Measuring the motility properties of particles—or cells—undergoing random motion is heavily dependent on the sampling rate. Indeed, since the cells can move back and forth, we expect the net distance traveled Δ**r**(*τ*) over a time *τ* to be sub-linear. By measuring the average distance traveled for different lag times we extract the experimental dependence in Supplementary Fig. 2c, d. We find that the distance traveled scales as a power law with an exponent smaller than 1 (which corresponds to ballistic motion): Δ**r**(*τ*) ~ *τ*^0.6^. This translates to an experimental average velocity which scales as: **v**(*τ*) ~ *τ*^−1.4^.

#### Testing the statistical power of the different experimental observables on the killing

In the same chip we can record heterogeneous spheroid outcomes; some spheroids fragment very fast (complete destruction at 8 h), whereas others are left unscathed at 14 h (see Fig. 4d).

Since the secondary droplet contains variable CTL numbers dependent on the initial cell concentration, this leads to a range of CTL numbers in the main droplet after droplet fusion (see Supplementary Fig. 1b). In addition to the number of CTLs in the droplet *N*, we record several other features: the first to the fourth contact times (*t*_1_ to *t*_4_, indicating the moment at which the number of cells on the spheroid goes above 1 to 4 cells), the spheroid projected area and the maximum number of CTLs on the spheroid within the 14 h of the experiment duration *n*.

We conduct the test with a generalized linear model^42^. The total observation sample size is of 96 events. This test enables us to study the influence and statistical power of each variable on visible spheroid fragmentation at 14 h. We see that only two variables have *p*-value below 0.05: the total number of CTLs in the well (*p*_*n*_ = 0.019, *β*_*n*_ = 1.84) and the maximum number of CTLs on the spheroid during the time-lapse (*p*_*N*_ = 0.028, *β*_*N*_ = 1.56). These two measures are very correlated (Supplementary Fig. 4); a high number of CTLs in the droplet increases the chances of having a high number of CTLs on the spheroid. The regression coefficients *β*_*n*_ and *β*_*N*_ were positive in both cases, confirming the positive correlation between these variables and the fragmentation probability. Interestingly, the other variables do not significantly predict the final spheroid state despite varying levels of correlation with spheroid death.

#### Inferring Γ_frag_ from experimental data

At each time frame, the CTLs on the spheroid can either leave the spheroid, cause a fragmentation event or do nothing. Conversely, CTLs in the gel can either stay in the gel or attach to the spheroid. We consider the killing to be independent of the attachment/detachment process and only to depend on the number of CTLs on the spheroid at any given time. From the point of view of the spheroid, we can therefore model the killing process as a Bernoulli process where the probability of seeing a fragmentation event only depends on the number *n* of CTLs on it at that time, and of a fragmentation rate Γ_frag_. Γ_frag_ is the probability that the *n* CTL(s) on the spheroid cause a fragmentation during a single time-interval Δ*t*. An implicit assumption of this model is that each time-step is independent: the probability of fragmenting during a time-interval only depends on the number of CTLs on the spheroid at the time and not on the previous CTL actions.

From the experimental fragmentation times and the observed evolution of the number of CTLs on the spheroid, we can infer the evolution of Γ_frag_ as a function of *n*. The probability that the first fragmentation event happens at time *T*_frag_ after exactly *k* time-steps is a function of Γ_frag_ and of the number **n** of CTLs on the spheroid as a function of time. **n** in bold font is an uni-dimensional vector where the *i*-th component is the number of CTLs on the spheroid at time *i*: *n*_*i*_. Since we suppose each time-step to be independent of the previous ones, the probability becomes:

4 $$P({T}_{{{{frag}}}}=k| {{{{{{{\bf{n}}}}}}}},{{{\Gamma }}}_{{{{frag}}}})={{{\Gamma }}}_{{{{frag}}}}({n}_{k})\mathop{\prod }\limits_{t}^{k-1}\big(1-{{{\Gamma }}}_{{{{frag}}}}({n}_{t})\big).$$

Since each droplet is independent, we can also estimate the probability of observing the set of fragmentation times ${\{{T}_{{{{frag}}}}\}}_{i}$:

5 $$P({\{{T}_{{{{frag}}}}\}}_{i}| {\{{{{{{{{\bf{n}}}}}}}}\}}_{i},{{\Gamma }})=\mathop{\prod}\limits_{i}P({T}_{{{{frag}}},i}| {{{{{{{{\bf{n}}}}}}}}}_{i},{{{\Gamma }}}_{{{{frag}}}}).$$

Now we can use maximum-likelihood estimation (ML) to extract the value of Γ_frag_ that maximizes the probability to observe the experimental fragmentation times given the experimentally observed time-series **n**. In short, we calculate Γ_frag_:

6 $${{{\Gamma }}}_{{{{{{{{\rm{frag}}}}}}}}}=\arg \mathop{\max }\limits_{{{{\Gamma }}}_{{{{{{{{\rm{frag}}}}}}}}}}P({\{{T}_{{{{frag}}}}\}}_{i}| {\{{{{{{{{\bf{n}}}}}}}}\}}_{i},{{{\Gamma }}}_{{{{{{{{\rm{frag}}}}}}}}}).$$

After some algebra this yields the following value for Γ_frag_(*n*):

7 $${{{\Gamma }}}_{{{{{{{{\rm{frag}}}}}}}}}(n)=\frac{{K}_{{{{Death}}},n}}{{K}_{{{{Death}}},n}+{K}_{n}},$$

where *K*_Death,*n*_ is the number of times we observe a fragmentation event happening with exactly *n* CTLs on the spheroid, and *K*_*n*_ the total number of times *n* CTLs are on the spheroid before any fragmentation event. We can verify that if there never is any death observed with *n* CTLs on the spheroid, then *K*_Death,*n*_ = 0 and Γ_frag_(*n*) = 0 too. Reversely, if there is a fragmentation event as soon as *n* cells are on the spheroid, then *K*_*n*_ = 0 and *K*_Death,*n*_ > 0, which gives Γ_frag_(*n*) = 1.

#### Probabilistic modeling of cooperative vs. independent killing of B16 cells on the spheroid by CTLs

In Fig. 5g, we show that the probability of a spheroid to undergo a fragmentation event increases with the number of CTLs on the spheroid surface. Furthermore, the maximum T cell number on the spheroid is a key variable predicting the final spheroid state. However, we do not know if this increase in Γ_frag_ is the result of the accumulation of independent random events or if it is the signature of cooperation between T cells.

We consider that the probability for a given T cell to cause fragmentation is independent of the presence of the other T cells. In quantitative terms, the probability of a fragmentation event occurring within the independent cell hypothesis is given by:

8 $${{{\Gamma }}}_{{{{{{{{\rm{frag}}}}}}}}}(n)=1-{\left(1-\rho \right)}^{n},$$

where the total number of CTLs on the spheroid is given by *n* and *ρ* is the probability of a single CTL to kill during a time-interval Δ*t*. Fitting Eq. (8) to the experimental measurements we see that the model does not accurately reflect experimental results (see Fig. 5g).

A first possible improvement for the independent CTL hypothesis is proposed by ref. ^33^ and consists of accounting for the heterogeneous nature of the CTL population. Indeed, it is well-known that CTLs differ widely in efficacy^43^. In order to test this hypothesis, we now model the CTL population as composed of independent cells, but the probability of being a fragmentation-causing CTL *ρ* is now itself drawn from a probability distribution ${{{{{{{\mathcal{K}}}}}}}}$ of probability density *f*. The average value of *ρ* is given by $\langle \rho \rangle =\int \rho f(\rho )\,d\rho$. We choose to not specify ${{{{{{{\mathcal{K}}}}}}}}$ to preserve generality. Then Eq. (8) becomes:

9 $${{{\Gamma }}}_{{{{{{{{\rm{frag}}}}}}}}}(n)	= \,1-{\mathbb{E}}\left[\mathop{\prod }\limits_{i = 1}^{n}\left(1-{\rho }_{i}\right)\right]\\ 	 = \,1-{\left(\int\left(1-\rho \right)f(\rho )d\rho \right)}^{n}\\ 	 = \,1-{\left(1- \langle \rho \rangle \right)}^{n}.$$

Provided that the statistics are sufficient, the result is similar to that in Eq. (8). Thus, the expected fragmentation probability is going to display a parabolic profile, whatever the heterogeneity profile of the CTL population is. Therefore the initial CTL population heterogeneity cannot explain the fragmentation profile recovered experimentally. Thus the CTLs cooperate to cause the fragmentation of the spheroid.

#### Estimating the attachment and detachment rates (*λ*_in_ and *λ*_out_)

A dedicated imaging and analysis pipeline was developed for this project in order to segment the spheroids and detect the CTLs in the focal plane within the droplets. The spheroid detection outputs a mask that corresponds to the spheroid position at every frame. Then, by detecting the CTLs that are in contact with this mask, it is possible to label each detected CTL as being on the spheroid or not. This information allows us to track individual arrival and leaving events on each spheroid. The high temporal resolution of the imaging (one image every 2 min) ensures that at most one event happens between two consecutive images (see Supplementary Movie 2).

The statistics of attachment and detachment are then used within a Markov chain model to estimate the attachment and detachment rates (*λ*_in_ and *λ*_out_) on the spheroid as a function of the number of CTLs on the spheroid. The evolution of these rates is then used to detect whether the attraction of the cells takes place through an active process or whether it is due to random chance.

Three hypotheses are required for the purpose of calculating *λ*_in_ and *λ*_out_: First, we suppose that the CTLs have had enough time to distribute themselves in the droplet before they begin to attach/detach. This is justified by noting that the average first contact time is large compared with the time to cross the droplet. Second, each of the attachment/detachment events occurs independently of other attachment/detachment events. Third, We hypothesize that the waiting times for each individual CTL arriving/detaching on the spheroid is exponentially distributed (i.e., the CTL is memory-less), and the waiting time parameter only depends on the number *n* of CTLs already on the spheroid. Finally, we only consider attachment and detachment events and not the movement of the CTLs in the droplet. Using these main hypotheses it is now possible to derive the model itself.

Consider a droplet with *N* CTLs and a single spheroid in it. The spheroid can be in one of *N* + 1 states, each state representing the number of CTLs on the spheroid (i.e., if the spheroid is in state *n*, then it has *n* CTLs on it). The experimental time series of discrete observations is denoted ${\{{n}_{t}\}}_{t}$ where *n*_*t*_ is the number of CTLs on the spheroid at time *t*. The set of transition probabilities is written ${{{{{{{\bf{p}}}}}}}}={\{{{{{{{{{\bf{p}}}}}}}}}_{k,l}\}}_{k,l}$, where **p**_*k*,*l*_ is the probability to go from *k* to *l* CTLs on the spheroid.

The system and its evolution can be described as a Markov chain. We call ${\mathbb{P}}\left({n}_{t}| {n}_{t-1}\right)$ the probability of having *n*_*t*_ CTLs on the spheroid at time *t* knowing that there were *n*_*t*−1_ CTLs on it at *t* − 1. ${\mathbb{P}}\left({\{{n}_{t}\}}_{t}\right)$ is the probability of observing the time-series ${\{{n}_{t}\}}_{t}$. By defining *a*_*k*,*l*_ as the number of transitions from *k* to *l* CTLs on the spheroid, it is possible to link ${\mathbb{P}}\left({\{{n}_{t}\}}_{t}\right)$ to the attachment and detachment statistics. Indeed, by virtue of the chain rule we have:

10 $${\mathbb{P}}\left({\{{n}_{t}\}}_{t}| {{{{{{{\bf{p}}}}}}}}\right)=\mathop{\prod}\limits_{t}{\mathbb{P}}\left({n}_{t}| {n}_{t-1}\right)=\mathop{\prod}\limits_{k,l}{{{{{{{{\bf{p}}}}}}}}}_{k,l}^{{a}_{k,l}}.$$

Equation (10) then allows us to infer the transition probabilities **p**. We conduct a maximum-likelihood estimate of **p**, under the constraint that the total out-going probability must add up to one (i.e., ∑_*k*_**p**_*k*,*l*_ = 1). This yields:

11 $${{{{{{{{\bf{p}}}}}}}}}_{k,l}=\arg \mathop{\max }\limits_{{{{{{{{{\bf{p}}}}}}}}}_{k,l}}\log \left({\mathbb{P}}\left({\{{n}_{t}\}}_{t}| {{{{{{{\bf{p}}}}}}}}\right)\right)=\frac{{a}_{k,l}}{{\sum }_{j}{a}_{k,j}}.$$

In practice, **p**_*k*,*l*_ is calculated from Eq. (11) by dividing the number of observed transitions from *k* to *l* cells by the total number of transitions starting from *k* cells (i.e., the number of times there are *k* cells on the spheroid).

Until now, we have studied the problem from the viewpoint of the spheroid: the probabilities **p**_*k*,*l*_ do not take into account the combinatorial aspect of the transitions (i.e., there are often more than one CTL in the gel that may attach to the spheroid). It is therefore important to look at the problem from the viewpoint of the individual CTLs and obtain the individual T cell attachment probabilities. Supposing that they attach independently of the other CTLs in the gel, we can calculate the individual probability for each CTL to attach to the spheroid, *p*_in_(*n*), as:

12 $${p}_{{{{{{\rm{in}}}}}}}(n)=1-{(1-{{{{{{{{\bf{p}}}}}}}}}_{n,n+1})}^{\frac{1}{N-n}}.$$

Given the exponential distribution of waiting times for attachment of the CTLs, the probability that a CTL arrives on a spheroid with *n* CTLs on it during a time-interval Δ*t* is ${p}_{{{{{{\rm{in}}}}}}}(n)=1-\exp \left(-{\lambda }_{{{{{{\rm{in}}}}}}}(n)\,{{\Delta }}t\right)$. Then the transition rates can be written as

13 $${\lambda }_{{{{{{\rm{in}}}}}}}(n)=-\frac{\log \left(1-{p}_{{{{{{\rm{in}}}}}}}(n)\right)}{{{\Delta }}t}.$$

Finally combining Eqs. (11), (12), and (13), provides a formula for *λ*_in_(*n*):

14 $${\lambda }_{{{{{{\rm{in}}}}}}}(n)=-\frac{\log (1-{{{{{{{{\bf{p}}}}}}}}}_{n,n+1})}{(N-n){{\Delta }}t}.$$

This formula allows us to directly estimate the attachment rate from the experimentally measured attachment events. To estimate the uncertainty of our estimations we conduct a bootstrapping scheme, where we randomly select droplets, pool them together and calculate the hitting parameters for this experiment subset. We then repeat the procedure over multiple subsets (typically 50) to get a distribution of arrival rates *λ*_in_(*n*), which enables us to calculate the mean and the variance of *λ*_in_ for each *n*. The code to conduct the bootstrapping procedure is freely available on the GitHub repository associated with this publication.

The process is repeated while counting leaving events to estimate the leaving rate *λ*_out_(*n*) as a function of the number of CTLs on the spheroid, also using Eq. (14). The evolution of *λ*_in_ and *λ*_out_ is shown in Fig. 3.

It is worth noting that the evolution of the number of CTLs on the spheroid can be described using a master equation:

15 $$\frac{\partial {\mathbb{P}}(n,t)}{\partial t}={{{{{{{{\bf{q}}}}}}}}}_{n-1,n}\,{\mathbb{P}}(n-1,t)+{{{{{{{{\bf{q}}}}}}}}}_{n+1,n}\,{\mathbb{P}}(n+1,t)-({{{{{{{{\bf{q}}}}}}}}}_{n,n+1}+{{{{{{{{\bf{q}}}}}}}}}_{n,n+1}){\mathbb{P}}(n,t),$$

where **q**_*k*,*l*_ = **p**_*n*,*n*+1_/Δ*t* is the transition rate from *k* to *l* CTLs on the spheroid. Knowing that for small values of **p**_*n*,*n*+1_, we have **p**_*n*,*n*+1_ ≈ (*N* − *n*)Δ*tλ*_in_(*n*), Eq. (15) can be rewritten as

16 $$\frac{\partial {\mathbb{P}}(n,t)}{\partial t}=	\ \,(N-n+1)\,{\lambda }_{{{{{{\rm{in}}}}}}}(n-1)\,{\mathbb{P}}(n-1,t)+(n+1)\,{\lambda }_{{{{{{\rm{out}}}}}}}(n+1)\,{\mathbb{P}}(n+1,t)\\ 	 -\left((N-n)\,{\lambda }_{{{{{{\rm{in}}}}}}}(n)+n\,{\lambda }_{{{{{{\rm{out}}}}}}}(n)\right){\mathbb{P}}(n,t).$$

The master equation would allow an analytical solution to obtain *λ*_in_ and *λ*_out_ if these parameters were constant for all values of *n*. Since this is not the case, obtaining them from Eq. (16) would require a numerical approach. Nevertheless, it is known that the master-equation and the Markov chain represent the same Markov process^44^, so both methods should lead to the same estimated parameters.

#### Relating the spheroid fate to the number of CTLs in the droplet

We know that due to a positive feedback loop, CTLs exhibit different accumulation rates depending on the number of CTLs on the target spheroid. We have estimated the experimental distribution of the hitting probabilities above. We also know that a certain number of CTLs on the spheroid are necessary for a high killing probability (Fig. 5). Combining these two components we have written an algorithm which reproduces the accumulation and fragmentation processes in silico.

First, we simulate the accumulation process. We define the number of CTLs on the spheroid *n* and the number of CTLs in the gel *n*_gel_. Thanks to the estimated parameters *λ*_in_ and *λ*_out_ we can calculate the attachment and detachment probabilities *p*_in_ and *p*_out_. From Eq. (13) we get the equation for *p*_in_: ${p}_{{{{in}}}}({\lambda }_{{{{{{\rm{in}}}}}}}(n),{{\Delta }}t)\approx 1-\exp ({n}_{{{{gel}}}}{\lambda }_{{{{{{\rm{in}}}}}}}(n){{\Delta }}t)$. We then do a Bernoulli draw with probability *p*_in_ (resp. *p*_out_) to simulate the attachment (resp. detachment) of T cells on the spheroid. Then, to simulate the fragmentation of the spheroid, we conduct a Bernoulli draw of parameter Γ_frag_(*n*)Δ*t* to determine whether or not the spheroid fragments at that time-step.

We repeat these two steps iteratively until the experiment is elapsed. This procedure gives us the simulated fate of the spheroid over the course of the experiment. We have access to the evolution of the number of T cells on the spheroid with time, the status of the spheroid (fragmented or intact) and the first fragmentation time. By re-running this procedure 50 times for a given number of CTLs in the droplet *N* we get a simulated fragmentation probability that is presented in Fig. 6d.

### Reporting summary

Further information on research design is available in the Nature Research Reporting Summary linked to this article.

## Supplementary information

Supplementary Information

Description of Additional Supplementary Files

Supplementary Movie 1

Supplementary Movie 2

Supplementary Movie 3

Supplementary Movie 4

Supplementary Movie 5

Supplementary Software 1

Reporting Summary

## Data Availability

All raw data to reproduce the graphs in the figures are available with the article. Any further data required will be provided upon request. Source data are provided with this paper.

## Source data

Source Data

## Code availability

The code to extract spheroid and CTL positions from the image data is available at the following address: https://github.com/BaroudLab/CTL_tracking_in_droplets. Data processing and data analysis codes are available at this address: https://github.com/BaroudLab/CTL_analysis.
