# Supplementary material for: High resolution microfluidic assay and probabilistic modeling reveal cooperation between T cells in tumor killing
Source: Nat Commun. 2022 Jun 3;13:3111. doi: 10.1038/s41467-022-30575-2 (PMC9166723; doi:10.1038/s41467-022-30575-2)
Supplement: Supplementary file 1 — Supplementary Information [file 41467_2022_30575_MOESM1_ESM.pdf]

High resolution microfluidic assay and probabilistic modelling reveal cooperation between T cells in tumour killing

Gustave Ronteix,[1,2,*] Shreyansh Jain,[1,2,*] Christelle Angely,[1,2] Marine Cazaux,[3] Roxana Khazen,[3] Philippe Bousso,[3,†] and Charles N. Baroud[1,2,†]

[1]*Physical microfluidics and Bioengineering, Institut Pasteur, 25-28 Rue du Dr. Roux, Paris, France*

[2]*LadHyX, CNRS, Ecole polytechnique, Institut polytechnique de Paris, 91120, Palaiseau, France*

[3]*Dynamics of Immune Responses Unit, Equipe Labellisée Ligue Contre le Cancer, Institut Pasteur, Universite de Paris, INSERM U1223, 75015 Paris, France*

(Dated: March 7, 2022)

* Equal contributions

† Corresponding authors.

philippe.bousso@pasteur.fr, charles.baroud@pasteur.fr

I. SUPPLEMENTARY FIGURES

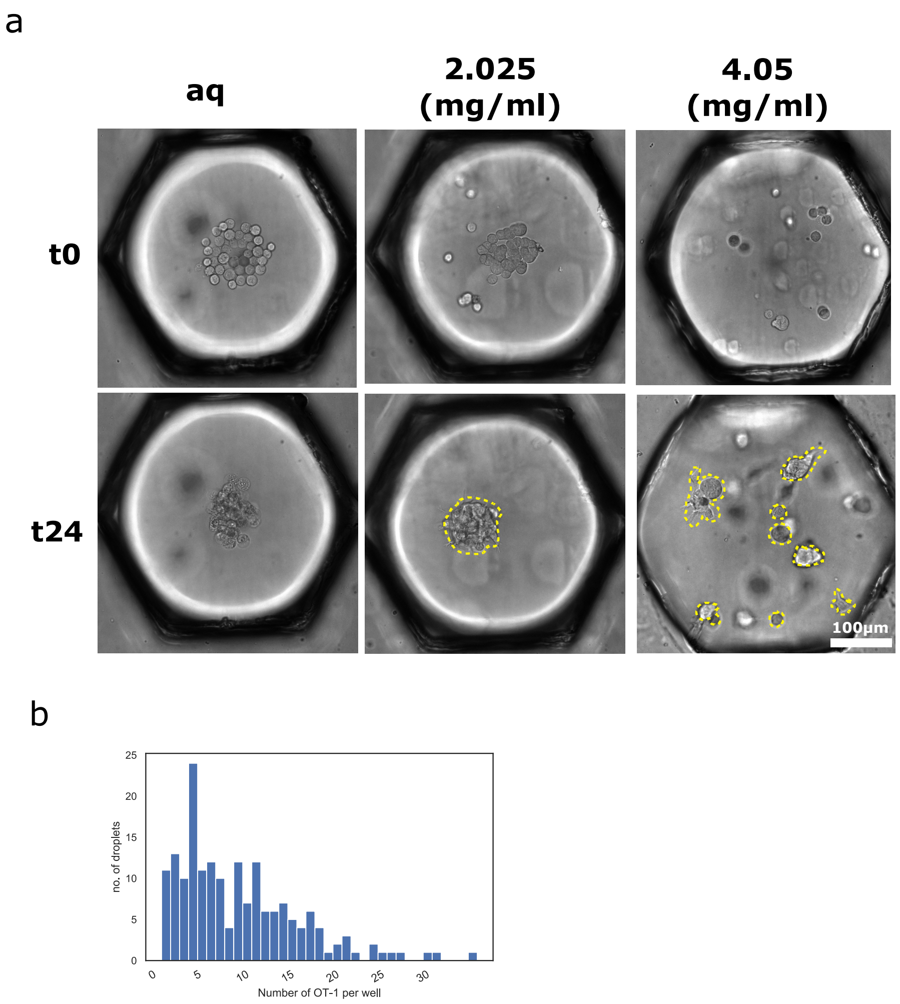

Supplementary Fig. 1. **a** Representative spheroids for different Matrigel concentrations at t_0 and $t_0 + 24h$ **b** Distribution of the number of CTLs per droplet $(n = 151)$

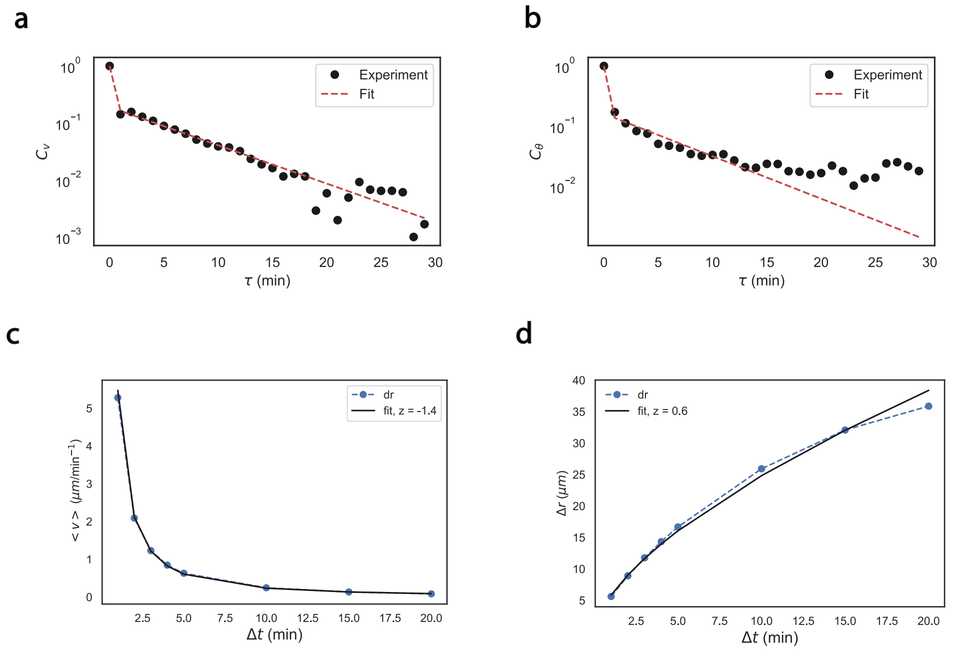

Supplementary Fig. 2. Velocity (**a**) and velocity-vector orientation (**b**) auto-correlation as a function of time. The experimental data (black dots) is fitted by a sum of two exponentials (dashed red line) in both cases. **c** Measured velocity as a function of the time-interval between two successive position measurements. The velocity scales as a power law. **d** Measured traveled distance as a function of the time-interval between two successive position measurements.

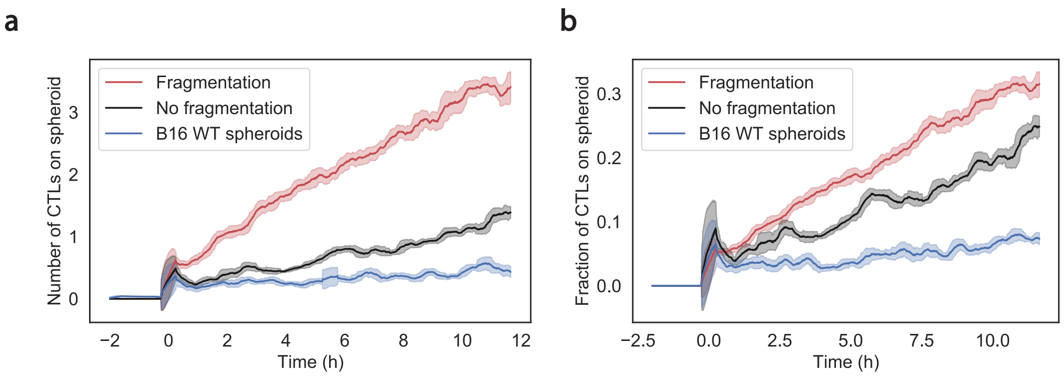

Supplementary Fig. 3. **a** Number of CTLs on the spheroid as a function of time for fragmenting spheroids (red), non-fragmenting spheroids (black) and with WT CTLs as a control (blue) **b** Fraction of CTLs on the spheroid as a function of time for fragmenting spheroids (red), non-fragmenting spheroids (black) and with WT CTLs as a control (blue)

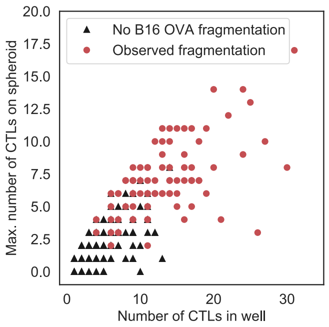

Supplementary Fig. 4. Spheroid fate as a function of the number of CTLs per droplet and the maximum number of detected CTLs on the spheroid.
