# Supplementary material for: High resolution microfluidic assay and probabilistic modeling reveal cooperation between T cells in tumor killing
Source: Nat Commun. 2022 Jun 3;13:3111. doi: 10.1038/s41467-022-30575-2 (PMC9166723; doi:10.1038/s41467-022-30575-2)
Supplement: Supplementary file 2 — Description of Additional Supplementary Files [file 41467_2022_30575_MOESM2_ESM.pdf]

Description of Additional Supplementary Files

File Name: Supplementary Movie 1
Description: **T cells find and attack cancer spheroids**
Montage of experiments showing T cells exploring matrigel droplets, finding spheroids, and attacking them. The spheroids display visible fragmentation in some cases, but not in others.

File Name: Supplementary Movie 2
Description: **T cells reaching spheroid**
Upon contact with the spheroid, T cells (green) change their motility mode and begin to explore the surface of the spheroid. Left movie shows bright-field images. Right movie shows edge-detection to highlight moment when cell is on spheroid vs. off spheroid.

File Name: Supplementary Movie 3
Description: **Dynamics of OVA-B16 vs. WT-B16 cases**
T cell accumulation on the spheroids is specific to the OVA-expressing B16 cells. Accumulation is only seen in the OVA expressing spheroids (left), while T cells do not attach nor accumulate on WT cells (right).

File Name: Supplementary Movie 4
Description: **T cells can leave OVA-B16 spheroids**
T cells can also leave OVA-expressing spheroids and migrate back into Matrigel matrix.

File Name: Supplementary Movie 5
Description: **Fragmentation is associated with Caspase 3/7 signal**
Simultaneous views of CTLs (green), Caspase 3/7 (red), and bright field views. The apparition of Caspase signal is well correlated with the adhesion of CTLs and the fragmentation of the B16 cells.

File Name: Supplementary Software 1
Description: Image analysis module and data analysis scripts for the quantitative analysis of CTL accumulation on spheroids.
