# Supplementary material for: High resolution microfluidic assay and probabilistic modeling reveal cooperation between T cells in tumor killing
Source: Nat Commun. 2022 Jun 3;13:3111. doi: 10.1038/s41467-022-30575-2 (PMC9166723; doi:10.1038/s41467-022-30575-2)
Supplement: Supplementary file 9 — Reporting Summary [file 41467_2022_30575_MOESM9_ESM.pdf]

nature research

Corresponding author(s): Philippe Bousso, Charles Baroud

Last updated by author(s): Mar 4, 2022

Reporting Summary

Nature Research wishes to improve the reproducibility of the work that we publish. This form provides structure for consistency and transparency in reporting. For further information on Nature Research policies, see our Editorial Policies and the Editorial Policy Checklist.

Statistics

For all statistical analyses, confirm that the following items are present in the figure legend, table legend, main text, or Methods section.

n/a	Confirmed	
☐	☒	The exact sample size (*n*) for each experimental group/condition, given as a discrete number and unit of measurement
☐	☒	A statement on whether measurements were taken from distinct samples or whether the same sample was measured repeatedly
☐	☒	The statistical test(s) used AND whether they are one- or two-sided<br>*Only common tests should be described solely by name; describe more complex techniques in the Methods section.*
☒	☐	A description of all covariates tested
☐	☒	A description of any assumptions or corrections, such as tests of normality and adjustment for multiple comparisons
☐	☒	A full description of the statistical parameters including central tendency (e.g. means) or other basic estimates (e.g. regression coefficient) AND variation (e.g. standard deviation) or associated estimates of uncertainty (e.g. confidence intervals)
☐	☒	For null hypothesis testing, the test statistic (e.g. *F*, *t*, *r*) with confidence intervals, effect sizes, degrees of freedom and *P* value noted<br>*Give P values as exact values whenever suitable.*
☒	☐	For Bayesian analysis, information on the choice of priors and Markov chain Monte Carlo settings
☒	☐	For hierarchical and complex designs, identification of the appropriate level for tests and full reporting of outcomes
☒	☐	Estimates of effect sizes (e.g. Cohen's *d*, Pearson's *r*), indicating how they were calculated

Our web collection on statistics for biologists contains articles on many of the points above.

Software and code

Policy information about availability of computer code

Data collection	Raw data collection was done using imaging software Nikon Elements (version 5.11.01, Build 1367)
Data analysis	1) Image analysis Python (version 3) based code to extract CTL positions from the original images (see code at https://github.com/BaroudLab/CTL_tracking_in_droplets)<br>2) Data processing and data analysis codes (available at https://github.com/BaroudLab/CTL_analysis)<br>3) custom made Macro is used in Imagej (version 1.53 f51) to analyze the spheroid size distribution.

For manuscripts utilizing custom algorithms or software that are central to the research but not yet described in published literature, software must be made available to editors and reviewers. We strongly encourage code deposition in a community repository (e.g. GitHub). See the Nature Research guidelines for submitting code & software for further information.

Data

Policy information about availability of data

All manuscripts must include a data availability statement. This statement should provide the following information, where applicable:

- Accession codes, unique identifiers, or web links for publicly available datasets
- A list of figures that have associated raw data
- A description of any restrictions on data availability

All raw data to reproduce the graphs in the figures are supplied. Any further data required can be supplied upon request.

Field-specific reporting

Please select the one below that is the best fit for your research. If you are not sure, read the appropriate sections before making your selection.

☒ Life sciences ☐ Behavioural & social sciences ☐ Ecological, evolutionary & environmental sciences

For a reference copy of the document with all sections, see nature.com/documents/nr-reporting-summary-flat.pdf

Life sciences study design

All studies must disclose on these points even when the disclosure is negative.

Sample size	Throughout the study, averages over multiple data points were performed and the results were successfully reproduced. Sample sizes (n) are indicated at the relevant location in manuscript. We performed experimental replicates with total of 84 droplets for B16-OVA experiments and 54 droplets for B16-WT experiments.
Data exclusions	No data were excluded for analysis.
Replication	The data for the B16-Ova originate from 3 independent microfluidic chips. The data for the B16-WT originate from 2 independent microfluidic chips.
Randomization	Randomization is not applicable for single cell studies, it is used in clinical studies.
Blinding	Blinding is not applicable for single cell studies, it is used in clinical studies.

Reporting for specific materials, systems and methods

We require information from authors about some types of materials, experimental systems and methods used in many studies. Here, indicate whether each material, system or method listed is relevant to your study. If you are not sure if a list item applies to your research, read the appropriate section before selecting a response.

Materials & experimental systems

n/a	Involved in the study	
☒	☐	Antibodies
☐	☒	Eukaryotic cell lines
☒	☐	Palaeontology and archaeology
☐	☒	Animals and other organisms
☒	☐	Human research participants
☒	☐	Clinical data
☒	☐	Dual use research of concern

Methods

n/a	Involved in the study	
☒	☐	ChIP-seq
☒	☐	Flow cytometry
☒	☐	MRI-based neuroimaging

Eukaryotic cell lines

Policy information about cell lines

Cell line source(s)	B16 melanoma cells (B16-WT and B16-Ova) were kindly provided by Claude Leclerc (Institut Pasteur).
Authentication	B16 (B16.F0) melanoma cells previously authenticated by ATCC (see vendor website). The phenotype of the cell lines used in the study was checked after thawing of the cells.
Mycoplasma contamination	Cells were tested negative for mycoplasma contamination (using Venor™ GeM Mycoplasma Detection Kit from MINERVA bio labs)
Commonly misidentified lines (See ICLAC register)	No commonly misidentified cell lines were used.

Animals and other organisms

Policy information about studies involving animals; ARRIVE guidelines recommended for reporting animal research

Laboratory animals	Male , Rag1-/- OT-I TCR, 6-8-week-old. The mice were bred and managed by Institut Pasteur's animal facility with a central air conditioning equipment which maintains constant temperature of 22 ± 2°C. Air is renewed at least 20 times per hour in animal rooms. Fluorescent light is provided with a 14:10 h light:dark cycle. Humidity is monitored but not controlled and in the range of 25-65%.

Wild animals	The study did not involve any wild animals
Field-collected samples	The study did not involve any collected from the field
Ethics oversight	All animal studies were approved by the Institut Pasteur Safety Committee in accordance with French and European guidelines (CETEA 2017-0038)

Note that full information on the approval of the study protocol must also be provided in the manuscript.
